# Pattern and perception of wellbeing, quality of work life and quality of care of health professionals in Southwest Nigeria

**DOI:** 10.1186/s12913-022-08808-3

**Published:** 2022-11-22

**Authors:** Oluwagbohunmi A. Awosoga, Nse A. Odunaiya, Olufemi O. Oyewole, Michael O. Ogunlana, Chidozie E. Mbada, Ogochukwu K. Onyeso, Opeyemi M. Adegoke, Ayomikun F. Ayodeji, Adesola C. Odole

**Affiliations:** 1 Faculty of Health Sciences, University of Lethbridge, Alberta, Canada; 2 Department of Physiotherapy, College of Medicine, University of Ibadan, Ibadan, Nigeria; 3 Department of Physiotherapy, Olabisi Onabanjo University Teaching Hospital, PMB 2001, Sagamu, Nigeria; 4 College of Health Sciences, University of KwaZulu-Natal, Private Bag X54001, Durban, South Africa; 5 Department of Physiotherapy, Federal Medical Centre, Abeokuta, Nigeria; 6 Department of Health Professions, Manchester Metropolitan University, Manchester, UK

**Keywords:** Quality of life, Quality of work life, Personal wellbeing, Quality of care, Health professionals, Nigeria

## Abstract

**Background:**

Personal wellbeing (PW) including quality of life and work life is a very complex concept that influences health professionals’ commitment and productivity. Improving PW may result in positive outcomes and good quality of care. Therefore, this study aimed to assess the pattern and perception of wellbeing, quality of work life (QoWL) and quality of care (QoC) of health professionals (HPs) in southwest Nigeria.

**Methods:**

The study was a convergent parallel mixed method design comprising a cross-sectional survey (1580 conveniently selected participants) and a focus group interview (40 purposively selected participants). Participants’ PW, quality of life (QoL), QoWL, and QoC were assessed using the PW Index Scale, 5-item World Health Organization Well-Being Index, QoWL questionnaire, and Clinician QoC scale, respectively. The pattern of wellbeing, QoWL and quality of care of HPs were evaluated using t-test and ANOVA tests. Binary regression analysis was used to assess factors that could classify participants as having good or poor wellbeing, QoWL, and quality of care of HPs. The qualitative findings were thematically analyzed following two independent transcriptions. An inductive approach to naming themes was used. Codes were assigned to the data and common codes were grouped into categories, leading to themes and subthemes.

**Results:**

Of 1600 administered questionnaires, 1580 were returned, giving a 98.75% response rate. Only 45.3%, 43.9%, 39.8% and 38.4% of HP reported good PW, QoL, QoC and QoWL, respectively; while 54.7%, 56.1%, 60.2% and 61.6% were poor. There were significant gender differences in PW and QoC in favor of females. With an increase in age and years of practice, there was a significant increase in PW, QoWL and QoC. As the work volume increased, there was significant decrease in QoWL. Participants with master's or Ph.D. degrees reported improved QoWL while those with diploma reported better QoC. PWI and QoC were significantly different along the type of appointment, with those who held part-time appointments having the least values. The regression models showed that participant’s characteristics such as age, gender, designation, and work volume significantly classified health professionals who had good or poor QoC, QoWL, PW and QoL. The focus group interview revealed four themes and 16 sub-themes. The four themes were the definitions of QoC, QoWL, and PW, and dimensions of QoC.

**Conclusion:**

More than half of health professionals reported poor quality of work life, quality of life and personal wellbeing which were influenced by personal and work-related factors. All these may have influenced the poor quality of care reported, despite the finding of a good knowledge of what quality of care entails.

## Introduction

Health professionals (HPs) are a heterogeneous mix of health care service providers who work towards caring for patients as the most important goal, by applying the available best evidence domiciled within the respective professional contexts [1, 2]. According to the International Labour Organization (ILO) [3], health professionals encompass medical doctors, nursing and midwifery professionals, and allied health professionals (including dentists, pharmacists, occupational therapists, and physiotherapists). The density of each of the varied HPs differ among the workforce and also among the several regions and countries of the world. Generally, physicians and nurses/midwives account for the largest group of HPs worldwide [4, 5]. Despite the dearth of HPs in Nigeria, nurses account for the highest number in the health workforce. The National Human Resources for Health Strategic Plan [6] reports that a nurse: population ratio of 100:100,000 as compared to a doctor: population ratio of 30:100,000, a pharmacist: population ratio of 11:100,000, a physiotherapist: population ratio of 0.62:100,000 and an occupational therapist: population ratio of 0.10:100,000. This uneven and low distribution of HPs in the Nigerian health workforce is sometimes responsible for experienced nurses having to perform the role of doctors in some health institutions in the country. This has been shown to be responsible for low job satisfaction, heightened work stress, frustration, and burnout experienced by HPs, especially, in Nigeria [7].

Due to the occupational demands, health professionals are a high-risk group when it comes to stress, burnout, and lower levels of wellbeing, even during non-pandemic times [8]. However, the World Health Organization (WHO) requires that health care delivery should be effective, safe, people-centred care, timely, equitable, integrated, and efficient [9]. These in sum are referred to as quality of care [9]. Quality of care is “the extent to which healthcare services provided to individuals and patient populations improve desired health outcomes” and are consistent with current professional knowledge [10, 11].To improve quality of care, the WHO [9] encourages health professionals to participate in quality measurement and improvement with their patients; embrace a practice philosophy of teamwork; see patients as partners in the delivery of care; commit themselves to providing and using data to demonstrate the effectiveness and safety of the care.

Health professionals provide services differently because of the professional ‘heterogeneity’ of the group (e.g., physicians, nurses, physiotherapists etc.), as well as the diversity of patients’ needs [12]. Also, variabilities in factors, not limited to experience, individual abilities, and personalities [13] may influence the quality of care. As such, it is often difficult to reproduce consistent healthcare services. Personal factors of the provider and the patient, and factors pertaining to the healthcare organisation, healthcare system, and the broader environment affect healthcare service quality [14]. Personal factors of health care professionals are reflective of the wellbeing of such a person, either it is subjective or objectively measured, which is sometimes referred to as health status or health-related quality of life. Subjective appraisal of satisfaction with life by an individual in these domains of wellbeing is often referred to as personal wellbeing [15].

Health professionals work within healthcare organisation contexts and systems that are largely, a reflection of the quality of work life (QoWL) [16]. QoWL describes the broader job-related experience that is peculiar to individuals. Assessment of QoWL as a value-based process is aimed at enhancing the effectiveness of an organization and improving the quality of life at work for the employees [16]. Furthermore, assessment of QoWL represents an examination of influences of work on the goodness and meaning in life, as well as people's happiness and wellbeing [17]. According to Easton and Van Laar[18], QoWL can be evaluated in six domains: General Wellbeing (GWB), Home-Work Interface (HWI), Job-Career Satisfaction (JCS), Control at Work (CAW), Working Conditions (WCS), and Stress at Work (SAW). It is believed that QoWL of an individual is influenced by direct experience of work and by the direct and indirect factors that affect this experience [18]. Owing to its importance, there is substantial literature on wellbeing of health professionals [19, 20], while few studies have explored QoWL among these professionals and little or none on clinician quality of care. However, it appears there is a paucity of patterns and perceptions of these constructs along personal factors among Nigerian health professionals. Therefore, this study aimed to assess the pattern and perception of wellbeing, QoWL and quality of care of health professionals in southwest Nigeria. We further conducted qualitative focus group interviews to explain quantitative survey results in an explanatory sequential design.

## Methods

### Design

The cross-sectional study utilized a convergent parallel design of both qualitative and quantitative data collection (cross-sectional survey and focus group interview data collected at the same time and analyse separately). Our study was grounded on the concept of complementarity in mixed methods approach [21]. We chose a mixed methods design to further explore information that the quantitative survey alone may not have captured. The qualitative data will serve beyond the purpose of providing different data types and multiple sources of data, it will also serve the purpose of triangulation; which is an important decision in the attempt to establish the validity of constructs in research [22]. It was hypothesized that findings from both the survey and focus group interview would provide a rich understanding of the subject matter. Mixed Methods Article Reporting Standards was used to report the study [23].

### Participants

The participants for the study were health professionals from selected hospitals in southwest Nigeria. The designated health professionals were medical practitioners, nurses, pharmacists, physiotherapists, occupational therapists, medical laboratory scientists, radiographers, and others (dental therapists, clinical psychologists, and dietitians). In Nigeria’s context, medical practitioners refer to the heterogeneous group from the various specialties of medicine, as well as dentists. The rest of the health professionals are referred to as allied healthcare workers. The hospitals involved in this study were teaching/tertiary hospitals. To be included in the study, a hospital must (i) be a tertiary hospital (referral center for primary and secondary levels of care), (ii) be public-funded, (iii) have at least 300-bed capacity, (iv) and at least 500 employees. Four of the tertiary hospitals that met the inclusion criteria were selected. The hospitals are the Federal Medical Centre (FMC), Abeokuta and Olabisi Onabanjo University Teaching Hospital (OOUTH), Sagamu, both in Ogun State; Obafemi Awolowo University Teaching Hospital Complex (OAUTHC), Ile-Ife, Osun State; and the University College Hospital (UCH), Ibadan, Oyo State. UCH, OAUTHC, FMC and OOUTH have bed capacities of 850, 842, 400, and 354 with workforce of 3000, 1490, 2000, and 566 health professionals, respectively.

### Participant recruitment

A convenience sampling technique was used to recruit the designated health professionals for the quantitative survey. Participants were included in the quantitative study if they were (i) health care workers in any of the four selected hospitals, (ii) fluent in the English language, able and willing to complete the questionnaire, and (iii) granted informed consent. Prior to the study, sample size was determined using a standard formula [24], the calculation parameters were confidence level = 95%, proportion (*p*) = 0.5, error margin = 0.05, Z-score = 1.96 and population size for selected hospitals = 7056. The minimum sample size to power the study is 1200. The calculated minimum sample size was 1200; however, in anticipation of incomplete surveys and attrition, we distributed 1600 surveys of which only 1580 surveys were returned completed. Quantitative and qualitative data were collected between May and November 2021.

To explore peculiar experiences concerning perceptions of quality of care received at the public-funded hospitals selected in this study, a qualitative focus group interview of health professionals was carried out. A purposive sample of 8 to 12 designated health professionals were recruited from each of the selected hospitals. The participants were individuals at the middle or senior professional cadres, with a minimum of five years of working experience, who were considered to provide the best information on the concepts of discussion. A sample size of 30 is adjudged large enough for a qualitative study to allow the unfolding of a ‘new and richly textured understanding’ of the phenomenon under study [25]. In sum, 40 participants (8 nurses, 13 medical practitioners, 5 pharmacists, 9 physiotherapists and 5 medical laboratory scientists) were recruited for the qualitative aspect of the study. Eight participants were drawn from FMC, 12 from UCH and 10 each from OOUTH and OAUTHC. The participants in both quantitative and qualitative aspects of the study gave informed consent. The study was performed in accordance with the declaration of Helsinki and ethical approval was obtained from the institution review boards of the centres where the data were collected.

### Researcher description

The researchers were a multidisciplinary team of scholars with expertise in both quantitative and qualitative methodology. The moderator for the focus group interview was a female physiotherapist in academics who holds a Ph.D. degree with over 15 years of experience in qualitative methodology. The interviewer used her knowledge of hospital settings in Nigeria to probe the respondents further.

### Data collection

For the quantitative cross-sectional aspect of the study, standard tools were used to assess the construct of interest. The questionnaires that were used in the study were self-administered and were distributed through hand delivery.

### Wellbeing of health professional

The 5-item World Health Organization Wellbeing Index (WHO-5) [26] and Personal Wellbeing Index [27] were employed to assess the construct. The WHO-5 assesses subjective health-related wellbeing while Personal Wellbeing Index assesses subjective wellbeing through questions of satisfaction directed to people’s feelings about themselves.

***The 5-item World Health Organization Wellbeing Index*** ***(WHO-5)*** is a brief and generic global rating scale measuring subjective wellbeing. The WHO-5 which was derived from the WHO-10 [28] tool, has sufficient psychometric properties with responsiveness ranges from 0.77 to 1.00 and with average sensitivity and specificity of 0.86 and 0.75, respectively [29] and has been utilized in many settings [30–34]. The raw score from the tool was calculated by summing the scores from the five answers, which has a range between 0 and 25 (0: worst possible; 25: best possible quality of life). The scores obtained were transformed to obtain a percentage score ranging from 0 to 100, by multiplying the raw score by four. A percentage score of 0 represents the worst possible, whereas a score of 100 represents the best possible quality of life. The scores were dichotomized as poor < 70% and good ≥ 70%.

***Personal Wellbeing Index Scale (PWI)*** was used to measure Quality of life. The scale consists of 8 items. Each item corresponds to a quality of life domain of standard of living, health, achievement in life, relationships, safety, community-connectedness, future security and spirituality. The first seven domains are the core domains while the spirituality domain is optional. It is measured on an 11-point Likert scale ranging from zero to ten (0 = extremely dissatisfied, 10 = extremely satisfied). Higher scores reflect a higher level of satisfaction with each domain. The PWI was found a valid and reliable measure of life satisfaction for use in various populations with Cronbach’s alpha ranges from 0.88 to 0.92 [15, 35, 36]. The scores were converted to maximum obtainable scores of 100% and were dichotomized as poor < 75% and good ≥ 75%.

### Quality of work life

The quality of work life questionnaire by Van Laar et al. [37] was utilized in this study. The tool assesses job control and satisfaction, work conditions, general wellbeing, work-life balance, stress at work, and control at work. The questionnaire is based on a 5-point Likert scale from 0 (strongly disagree) to 4 (strongly agree) for positive items and it measures at the ordinal level. A test–retest reliability of 0.78 Cronbach's alpha was reported in a study by Shabaninejad et al. [38]. The scores were converted to maximum obtainable scores of 100% and were dichotomized as poor < 70% and good ≥ 70%.

### Quality of care

The assessment of quality of care was from health professional's perspective. Thus, the Clinician Quality of Care scale was employed [39]. It is a 22-item questionnaire consisting of two factors: Person-Centered Care and Discordant Care. It is assessed on 6-point Likert scale, 0 = never to 5 = always where clinicians are asked to report how frequently each item had occurred in the past six months. Internal consistency (Cronbach’s alpha ranges 0.74 – 0.86) and validity with measures related to satisfaction and the therapeutic relationship have been judged to be adequate [39]. The scores were converted to maximum obtainable scores of 100% and were dichotomized as poor < 75% and good ≥ 75%.

### Focus group interview

Four face-to-face focus group interviews were conducted in private, well ventilated, noiseless rooms without distraction. There was one focus group interview in each of the selected hospitals. Each interview consisted of a moderator, a note taker, and selected health professionals. An interview guide was utilized with questions, prompts, and guides provided by the moderator. The semi-structured interview questions were open-ended to ensure that participants expressed their views and shared their experiences about how quality of care was hindered by their wellbeing and quality of work life in their respective work settings. The interview was audio recorded and field notes were also taken during the interview. Each of the focus group interviews lasted for 90 min.

### Data analysis

The quantitative data analyses were completed using descriptive and inferential statistics. Descriptive statistics – frequency (percentage) and mean ± standard deviation were used to summarize the sociodemographic characteristics of the participants. For inferential statistics, the data was diagnosed and fixed for missing variables, and univariate and multivariate outliers; all data were normally distributed. Therefore, an independent samples t-test (a parametric test used in comparing differences between two levels of independent variable e.g. gender having a dependent variable with a scale/continuous/Normal level measurement) and one-way analysis of variance (ANOVA, a parametric test used in comparing differences between two or more levels of independent variable e.g. age range and designation having a dependent variable with a scale/continuous/Normal level measurement) were used to determine the mean differences in participants’ pattern of wellbeing, QoWL and quality of care across the sociodemographic characteristics. Binary logistic regression (forward Wald mode of entry) was used to determine the set of factors that best-classified participants into good or poor wellbeing, QoWL and quality of care. In a situation like our study, where multiple predictors were simultaneously tested, the Forward Wald (stepwise) approach was used to limit the final model to statistically significant predictors. The alpha value was set at 0.05. Statistical analyses were carried out using SPSS 27.0 version software (SPSS Inc., Chicago, Illinois, USA).

The qualitative findings were thematically analyzed by two independent transcriptionists (who transcribed the recording) and also used field notes. Codes were assigned to the data and common themes were described based on frequencies. The themes were derived from the data. An inductive approach to naming codes and themes were used. A Computer assisted qualitative data analysis software known as ATLAS.TI (version 9) software was used for the analysis.

## Results

 One thousand five hundred and eighty (1580) health professionals participated in the quantitative aspect of the study out of 1600 that were invited, giving a response rate of 98.75%. Most participants were females (982; 62.2%) who had bachelor’s degree (1076; 68.1%) with full-time employment (1380; 87.3%). The 40 focus group participants were gender-matched and most (87.5%) had ≥ 11 years of practice. More information on the participants’ characteristics is presented in Table 1.

**Table 1:** Respondents’ quality of life, work life, care, and personal wellbeing (*n* = 1580)

Parameter	*n* (%)	QoL	PWI	QoWL	CQoC
	**1580 (100%)**	**mean (SD)**	**mean (SD)**	**mean (SD)**	**mean (SD)**
**Gender**					
Female	982 (62.2)	61.73(21.84)	72.71(14.6)	65.9(10.55)	71.48(12.84)
Male	585 (37.0)	61.99(20.39)	69.78(14.52)	65.41(10.50)	68.02(12.29)
Total	1567 (99.2)	61.82 (21.31)	71.61 (14.63)	65.71 (10.53)	70.19 (12.74)
* t*-statistic (df)		-0.241 (1565)	3.841 (1565)	0.903 (1565)	5.250 (1565)
* p*-value		0.809	< 0.001*	0.367	< 0.001*
**Age group**					
20 – 29	303 (19.2)	61.73 (19.20)	69.00 (15.52)	64.61 (9.29)	69.14 (13.22)
30 – 39	618 (39.1)	61.04 (20.98)	69.55 (15.01)	64.49 (10.62)	68.46 (12.87)
40 – 49	367 (23.2)	60.99 (21.82)	73.61 (12.85)	66.45 (10.10)	71.49 (12.7)
50 – 59	211 (13.4)	66.23 (22.54)	77.06 (12.99)	69.44 (10.58)	72.67 (11.35)
60 – 69	6 (0.4)	66.67 (30.53)	81.04 (9.03)	73.89 (9.90)	77.12 (9.95)
Total	1505 (95.03)	61.92 (21.16)	71.53 (14.61)	65.72 (10.37)	69.96 (12.78)
* F*-statistic (df _1_, df _2_)		2.728 (4, 1500)	15.776 (4, 1500)	11.512 (4, 1500)	6.702 (4, 1500)
* p*-value		0.028*	< 0.001*	< 0.001*	< 0.001*
**Years in practice**					
0 – 2	512 (32.4)	61.98 (18.96)	68.95 (15.03)	64.27 (10.22)	69.23 (13.2)
3 – 5	328 (20.8)	62.51 (21.47)	70.81 (15.20)	64.82 (11.01)	67.89 (13.54)
6 – 10	263 (16.6)	62.58 (21.22)	72.43 (13.86)	65.48 (9.91)	69.47 (12.37)
≥ 11	471 (29.8)	60.99 (23.50)	74.61 (13.65)	68.06 (10.43)	73.07 (11.36)
Total	1574 (99.6)	61.89 (21.28)	71.61 (14.64)	65.72 (10.51)	70.14 (12.76)
* F*-statistic (df _1_, df _2_)		0.468(3, 1570)	13.101(3, 1570)	12.111(3, 1570)	13.094(3, 1570)
* p*-value		0.705	< 0.001*	< 0.001*	< 0.001*
**Education level**					
National Diploma	134 (8.5)	65.31 (21.25)	74.00 (14.42)	65.50 (12.06)	73.14 (13.60)
Bachelor (entry-level)	1076 (68.1)	61.56 (21.03)	70.80 (15.12)	64.87 (10.41)	69.36 (13.03)
Masters or Ph.D	359 (22.7)	62.53 (21.25)	72.90 (13.04)	68.22 (9.86)	71.17 (11.37)
Total	1569 (99.3)	62.10 (21.11)	71.55 (14.65)	65.69 (10.52)	70.10 (12.77)
* F*-statistic (df _1_, df _2_)		1.970 (2, 1566)	4.834 (2, 1566)	13.894 (2, 1566)	6.945 (2, 1566)
* p*-value		0.140	0.008*	< 0.001*	0.001*
**Designation**					
Nurse	570 (36.1)	63.77 (22.12)	74.48 (14.5)	66 (11.17)	72.31 (13.50)
Medical practitioner^a^	609 (38.5)	58.32 (20.97)	69.19 (14.62)	64.26 (10.34)	67.75 (12.55)
Pharmacist	145 (9.2)	62.29 (19.71)	69.91 (13.67)	67.43 (9.41)	67.03 (11.92)
Physiotherapist	120 (7.6)	67.67 (18.35)	70.91 (15.17)	67.35 (9.23)	72.15 (10.83)
Radiographer	7 (0.4)	64.57 (16.88)	65.89 (9.12)	62.74 (6.75)	70.78 (7.24)
Medical lab. scientist	108 (6.8)	64.22 (21.65)	73.66 (13.62)	68.65 (10.11)	73.98 (9.60)
Occupational therapist	10 (0.6)	60.80 (18.74)	67.75 (17.33)	61.25 (9.05)	77.18 (6.17)
Total	1569 (99.3)	61.83 (21.30)	71.59 (14.65)	65.69 (10.54)	70.18 (12.74)
* F*-statistic (df _1_, df _2_)		5.397(6, 1562)	7.621(6, 1562)	4.993(6, 1562)	10.783(6, 1562)
* p*-value		< 0.001*	< 0.001*	< 0.001*	< 0.001*
**Appointment**					
Full time	1380 (87.3)	62.1 (21.39)	72.11 (14.5)	65.76 (10.65)	70.83 (12.56)
Part time	187 (11.9)	60.25 (20.28)	67.72 (14.87)	65.25 (9.68)	64.90 (12.81)
Casual	10 (0.6)	64.00 (22.39)	75.63 (20.32)	68.75 (9.63)	70.91 (19.42)
Total	1577 (99.8)	61.89 (21.26)	71.61 (14.65)	65.72 (10.53)	70.13 (12.78)
* F*-statistic (df _1_, df _2_)		0.674(2, 1574)	7.830(2, 1574)	0.613(2, 1574)	18.171(2, 1574)
* p*-value		0.510	< 0.001*	0.542	< 0.001*
**Work schedule**					
Permanent morning and call duty	789 (49.9)	61.12 (20.24)	70.21 (14.22)	64.97 (10.39)	69.92 (11.61)
Shift duty	468 (29.6)	63.05 (22.52)	73.44 (14.82)	65.65 (10.72)	71.42 (13.27)
Shift and call duty	30 (1.9)	57.6 (19.27)	69.29 (13.01)	65.69 (8.58)	70.39 (15.48)
Permanent morning	289 (18.3)	62.54 (22.07)	72.73 (15.33)	67.89 (10.54)	68.64 (14.44)
Total	1576 (99.7)	61.89 (21.27)	71.61 (14.65)	65.72 (10.53)	70.14 (12.77)
* F*-statistic (df _1_, df _2_)		1.301(3, 1572)	5.685(3, 1572)	5.457(3, 1572)	3.000 (3, 1572)
* p*-value		0.272	0.001*	0.001*	0.03*
**Work volume per week**					
< 20 h	67 (4.2)	66.87 (18.18)	71.68 (18.54)	68.93 (11.35)	70.13 (12.22)
20–40 h	419 (26.5)	63.17 (21.02)	73.20 (13.93)	66.50 (9.26)	68.57 (13.60)
41–60 h	739 (46.8)	61.76 (21.69)	71.86 (14.25)	65.98 (10.88)	70.77 (12.85)
> 60 h	350 (22.2)	59.74 (21.10)	69.16 (15.23)	63.62 (10.77)	70.54 (11.51)
Total	1575 (99.7)	61.91 (21.28)	71.61 (14.65)	65.72 (10.54)	70.11 (12.77)
* F*-statistic (df _1_, df _2_)		2.929(3, 1571)	5.025(3, 1571)	7.739(3, 1571)	2.825(3, 1571)
* p*-value		0.033*	0.002*	< 0.001*	0.038*

*QoL* quality of life, *PWI* personal wellbeing index, *QoWL* quality of work life, *CQoC* clinician quality of care

^*^Test statistic = significant at *p* < 0.05

^a^Medical practitioner = physicians, surgeons, psychologist, dentist

Table 1 presents pattern of respondents’ quality of life, work life, care, and personal wellbeing. There was significant gender difference in PWI and QoC in favor of females. With increase in age, there was significant increase in QoL, PWI, QoWL and QoC. Also, with increase in years of practice, there was significant increase in PWI, QoWL and QoC. As the work volume increased, there was significant decrease in QoWL. Education level and work schedule had significant difference in PWI, QoWL and QoC. Those with master or Ph.D. degrees reported improved QoWL while those with diploma reported better QoC. PWI and QoC were significantly different along the type of appointment with those who held part time appointment having the least values.

Only 693 (43.9%) health professionals reported good QoL while 887 (56.1%) were poor. Poor and good PWI, QoWL, QoC were reported by 864(54.7%) and 716(45.3%); 973(61.6%) and 607(38.4%); 951(60.2%) and 629(39.8%) health professionals, respectively. Table 2 shows factors that best differentiate poor from good quality of life. Age, year of practice, designation, personal wellbeing and QoWL significantly predicted good QoL. With a unit increase in personal wellbeing and QoWL, there is thrice (OR = 3.318; *p* < 0.001) and twice (OR = 1.957; *p* < 0.001) the likelihood of reporting good QoL, respectively. Only nursing and medical practitioner designation significantly predicted good QoL. Medical practitioners were 0.466 less likely to report good QoL compared with nurses. The health professionals with ≥ 11 years of practice were 0.525 less likely to report good QoL compared with ≤ 2 years of practice. Those in age ranges 40–49 and 50–59 years were 1.961 and 2.403 more likely to report good QoL, respectively compared to age ranges 20–29 years.

**Table 2:** Factors that best differentiate poor from good quality of life (WHO-QoL)

Predictors	Regression Coefficients (B)	Odds ratio (β)	Wald	*P*-value
**Age (years)**				
20 – 29 (reference)			9.507	0.050*
30 – 39	0.362	1.436	3.658	0.056
40 – 49	0.673	1.961	7.263	0.007*
50 – 59	0.877	2.403	8.706	0.003*
60 – 69	1.249	3.486	1.436	0.231
**Gender** (reference = female)				
Male	0.279	1.322	3.740	0.053
**Years of practice**				
0–2 (reference)			12.788	0.005*
3–5	0.181	1.199	1.009	0.315
6–10	-0.268	0.765	1.586	0.208
≥ 11	-0.645	0.525	6.827	0.009*
**Designation**				
Nurse (reference)			32.411	< 0.001*
Medical practitioner	-0.763	0.466	20.401	< 0.001*
Pharmacist	-0.298	0.743	1.826	0.177
Physiotherapist	0.159	1.172	0.452	0.501
Radiographer	-0.226	0.798	0.053	0.819
Medical lab. Scientist	0.070	1.072	0.080	0.777
Occupational therapist	-0.560	0.571	0.597	0.440
Others	0.557	1.745	0.542	0.461
**Personal Wellbeing**	1.199	3.318	99.098	< 0.001*
**Quality of Work Life**	0.671	1.957	29.812	< 0.001*
**Constant**	**-1.136**	**0.321**	**41.371**	< 0.001*

Approach: Forward Wald binary logistic regression

Model summary: χ^2^ (17, *N* = 1567) = 256.12, *p* < 0.001. Nagelkerke *R*^2^ = 21.3%. Overall prediction success was also modest at 69.8%, with 60.0% of people with good QoL correctly classified and 77.3% of poor QoL classified

^*^ = statistic is significant at *p* < 0.05

Conversely, age, gender, work-related quality of life, QoC and QoL were factors that significantly classified health practitioners with good and poor personal wellbeing (Table 3). Males were 36% less likely to report good personal wellbeing compared to females (OR = 0.64; *P* < 0.001). Those in age ranges 50–59 years were 1.54 more likely to report good wellbeing compared to age ranges 20–29 years. With a unit increase in QoWL, QoC and QoL, there is 3.0, 1.5 and 3.4 more likelihood to report good personal wellbeing, respectively (Table 3).

**Table 3:** Factors that best classify health practitioners with good and poor personal wellbeing

Predictors	Regression Coefficients (B)	Odds ratio (β)	Wald	*P*-value
**Age (years)**				
20 – 29 (reference)			13.869	0.008*
30 – 39	-0.242	0.785	2.277	0.131
40 – 49	0.028	1.028	0.025	0.876
50 – 59	0.433	1.542	4.367	0.037*
60 – 69	0.221	1.247	0.048	0.826
**Gender** (reference = female)				
Male	-0.446	0.640	12.768	< 0.001*
**Quality of Work Life**	1.101	3.007	79.627	< 0.001*
**Clinician Quality of Care**	0.427	1.533	12.302	< 0.001*
**WHO-Quality of Life**	1.218	3.380	104.708	0.007*
**Constant**	**-1.146**	**0.318**	**56.099**	< 0.001*

Approach: Forward Wald binary logistic regression

Model summary: χ^2^ (8, *N* = 1580) = 326.725, *p* < 0.001. Nagelkerke *R*^2^ = 26.5%. Overall prediction success was also modest at 69.6%, with 62.5% of people with good personal wellbeing correctly classified and 75.5% of people classified under poor personal wellbeing

^*^ = statistic is significant at *p* < 0.05

Table 4 shows factors that best classify health practitioners with good and poor quality of work life. Age, education, designation, work volume, QoC, QoL and personal wellbeing significantly predicted QoWL. With a unit increase in QoC, QoL and personal wellbeing, there is 2.1, 2.0 and 3.0 more likelihood to report good QoWL, respectively. As the work volume increases, there is less likelihood to report good QoWL. Those with work volume of 20–40 h, 41–60 h and > 60 h were 54% (OR = 0.463; *P* = 0.013), 52% (OR = 0.480; *P* = 0.015) and 65% (OR = 0.350; *P* = 0.001) less likely to report good QoWL, respectively compared to < 20 h. Health professionals with master or Ph.D. degrees were twice likely to report good QoWL compared with diploma holders (OR = 2.255; *P* = 0.002). Those in age ranges 50–59 years were twice likely to report good QoWL compared to age ranges 20–29 years (OR = 2.144; *P* < 0.001).

**Table 4:** Factors that best classify health practitioners with good and poor quality of work life

Predictors	Regression Coefficients (B)	Odds ratio (β)	Wald	*P*-value
Age (years)				
20 – 29 (reference)			14.892	0.005*
30 – 39	0.234	1.264	1.844	0.175
40 – 49	0.141	1.151	0.515	0.473
50 – 59	0.763	2.144	11.164	< 0.001*
60 – 69	1.826	6.211	2.251	0.134
Education level				
National Diploma (reference)			12.087	0.002*
Bachelor	0.360	1.433	2.373	0.123
Masters or Ph.D	0.813	2.255	9.491	0.002*
Designation				
Nurse (reference)			13.474	0.061*
Medical practitioner	-0.108	0.898	0.426	0.514
Pharmacist	0.379	1.461	2.815	0.093
Physiotherapist	-0.350	0.705	1.947	0.163
Radiographer	0.877	2.404	0.688	0.407
Medical lab. scientist	0.210	1.233	0.706	0.401
Occupational therapist	-1.968	0.140	3.189	0.074
Others	0.839	2.315	1.385	0.239
Work volume per week				
< 20 (reference)			11.067	0.011*
20–40	-0.771	0.463	6.232	0.013*
41–60	-0.734	0.480	5.971	0.015*
> 60	-1.049	0.350	10.647	0.001*
Clinician Quality of Care	0.743	2.103	35.805	< 0.001*
WHO-Quality of Life	0.706	2.025	31.946	< 0.001*
Personal Wellbeing	1.115	3.048	79.071	< 0.001*
Constant	-1.572	0.208	16.678	< 0.001*

Approach: Forward Wald binary logistic regression

Model summary: χ^2^ (19, *N* = 1580) = 306.054, *p* < 0.001. Nagelkerke *R*^2^ = 25.4%. Overall prediction success was also modest at 72.5%, with 55.5% of people with good personal wellbeing correctly classified and 83.0% of people classified under poor personal wellbeing

^*^ = statistic is significant at *p* < 0.05

Table 5 presents factors that best classify health practitioners with good and poor quality of care. Factors that significantly predicted good quality of care are gender, designation, nature of appointment, work volume, personal wellbeing and QoWL. Males were 28% less likely to offer good quality of care compared with females (OR = 0.717; *P* = 0.018). Those in part time appointment were 37% less likely to offer quality of care compared to full time (OR = 0.628; *P* = 0.019). Those with work volume > 60 h were 1.891 more likely to offer good quality of care compared to < 20 h. With a unit increase in personal wellbeing and QoWL, there is 1.43 and 2.07 more likelihood to offer good quality of life (Table 5).

**Table 5:** Factors that best classify health practitioners with good and poor quality of care

Predictors	Regression Coefficients (B)	Odds ratio (β)	Wald	*P*-value
Age (years)				
20 – 29 (reference)			8.240	0.083
30 – 39	-0.209	0.811	1.668	0.196
40 – 49	0.142	1.153	0.643	0.423
50 – 59	-0.183	0.833	0.773	0.379
60 – 69	1.033	2.809	1.210	0.271
Gender (reference = female)				
Male	-0.333	0.717	5.571	0.018*
Designation				
Nurse (reference)			24.921	0.001*
Medical practitioner	-0.486	0.615	8.520	0.004*
Pharmacist	-0.751	0.472	11.030	0.001*
Physiotherapist	0.080	1.083	0.124	0.725
Radiographer	-0.868	0.420	0.582	0.446
Medical lab. scientist	0.039	1.039	0.027	0.870
Occupational therapist	1.237	3.446	2.940	0.086
Others	-0.687	0.503	0.912	0.340
Nature of appointment				
Full time (reference)			7.232	.027*
Part time	-0.466	0.628	5.527	.019*
Casual	0.818	2.266	1.367	0.242
Work volume per week				
< 20 (reference)			12.918	0.005*
20–40	0.103	1.109	0.122	0.727
41–60	0.498	1.646	2.994	0.084
> 60	0.637	1.891	4.242	0.039*
Personal Wellbeing	0.355	1.427	8.833	0.003*
Quality of Work Life	0.726	2.066	34.932	< 0.001*
Constant	-0.820	0.441	6.844	0.009*

Approach: Forward Wald binary logistic regression

Model summary: χ^2^ (19, *N* = 1580) = 149.198, *p* < 0.001. Nagelkerke *R*^2^ = 13.0%. Overall prediction success was modest at 65.4%, with 39.0% of people providing good quality of care correctly classified and 82.5% of people classified as providing poor quality of care

^*^ = statistic is significant at *p* < 0.05

Focus group interview revealed four themes and 16 sub-themes. The four themes are definition of quality of care, dimension of quality of care, definition of personal wellbeing and quality of work life.

### Theme 1: Definition of quality of care

Majority of the participants were able to provide their own meaning of quality of care. The four most recurring sub-themes were extracted as seen below:

#### Standard care

Three discussants from OAUTHC described quality of care to be the standard care that is given to a patient.

*“Quality of care simply means the required standard of care giving to particular clients in the institution.”* (Participant 21, female)

*“Quality of care could be optimal care or standard care offered to client.”* (Participant 23, male)

#### Satisfaction of patient and healthcare provider

Two discussants from FMC described quality of care to be a kind of care focused on satisfaction of both the patient and the healthcare provider. Two discussants from OAUTHC group agreed but argued that quality of care cannot be standard except the patient is satisfied with care received and the healthcare giver is also satisfied with giving care. Both parties must have satisfaction.

*“In my own opinion, I can describe quality of care to mean a kind of care I give such that I as a physiotherapist is satisfied, and my client also feels I have done the best I can in my capacity. So, the quality of care is perhaps related to both the caregiver as well as the person being care for. So, in my opinion, it stems from both ends, but as it relates to me as a health care provider, I can also say that quality of care could mean my own competence to be able to do what I’m supposed to do in that particular situation - how quality and how qualitative it is and how good it is – and also the satisfaction of the client.”* (Participant 15, female)

*“Well, for quality of care as we have said, there is a standard, there’s a minimum requirement that should be given to patients. So what comes to our mind is, has it been achieved? Has the environment and the equipment, and the personnel, are we equipped to offer that quality of care. And is everything actually okay with the caregiver, to provide that quality of care? To me that is very important.”* (Participant 25, male)

#### Timely, efficient, excellent, and equitable treatment

Two discussants from FMC stated that quality of care has to be timely, efficient, excellent, and equitable.

*“I'd like to add that it also has to do with efficiency, making sure that the quality of life of individuals improved because that is what quality care is. The goal is to improve their quality of life.”* (Participant 15, female)

Other discussants from OAUTHC and OOUTH agreed with them.

*“When you are talking about quality of care, I want to say care that is quality, valuable, worth the price, the care that is safe, that is timely, that you give at appropriate time.”* (Participant 4, female)

#### Holistic patient-centered care

The remaining participants from UCH and OOUTH defined quality of care as overall working conditions of the healthcare system centered on the patient.

*“When you are talking about quality, like in my own perspective, like we do in nursing practice, we want to talk about the patient-centered care. A care that the patient would appreciate.”* (Participant 4, female)

*“What I understand about quality of care is a care that is safe so that the patient would have the best and the whole community; a holistic thing for a universal health coverage.”* (Participant 2, female)

### Theme 2: Dimensions of quality of care

Different aspects that contribute to the wellbeing of the patient and the caregiver were visited by the discussants as important to quality care.

#### Adequate provision of care, materials, consumables, and manpower

Speaker 7 from OOUTH emphasized adequacy as something very important and a dimension to quality of care.

*“Talking about quality of care, we are health professionals, and we want to give adequate necessary care, a holistic care to our clients in all the departments in the teaching hospital.…….. the facility should be well equipped to be able to produce this care to pass it across to every client seeking help in this hospital. We are talking about manpower, we are talking about funding, we are talking about the orderliness of the environment; all the spheres. There should be adequacy in the provision made available to be able to care for all our patients.”* (Participant 7, female)

A Speaker from OAUTHC shared similar thoughts, however, he emphasized more on consistency in giving of care within the available resources.

*“It covers different level of operations. That’s what dimension is, because it entails what is obtainable at the primary center is different from what is obtainable at the tertiary sectors. Although there is quality of care that is acceptable in the primary care but what is more important is that excellent care given by the caregiver consistently within the available resources.*” (Participant 20, male)

#### Safe and conducive environment

Other discussants agreed that one of the dimensions to quality care is a conducive environment that is safe and offer holistic, timely and efficient care. A speaker from FMC pointed out the vitality of having conducive environment for both patient and the health caregiver.

*“Let me first start with the fact that to get quality of care by any patient, the patient actually will need a conducive environment to ensure that the quality is given. It is not only for the patient but for the caregiver. So, in one way or the other when the infrastructure is deficient both the caregiver and the patient suffers. The patient in the sense that they may not be able to get the best.”* (Participant 18, male)

Two other participants from the same group echoed similar view:

*“Like participant 2 rightly said I think the safety, being time bound, being efficient about it, having a good environment to work and also available facilities.”* (Participant 8, female)

#### Equal and equitable service

However, a speaker from FMC opined that whether that patient is rich or poor, young or old, politician or not, that person should be able to access the same type of care everywhere globally.

*“Well, as much as I agree with everybody, I believe the quality of care should also be related to the acceptable standard, not just for your own environment, except you have a standard by which you can rate the service you are giving, you may assess yourself to have done well and even the care – the person you have cared for could have assessed you to have done well. So, that is to say that there must be a standard to which you are relating whatever you are doing to; and the standard must be in terms of qualitative and quantitative measures. And that thing must be measurable and it must be reproducible –……reproduce what you have done and in terms of you being able to standardize it so that it gets to anyone in the world. The same thing that I’ve done for this person is what will be done not based on the locality that you find yourself or that particular period that you are carrying out the service to the individual.”* (Participant 12, female)

Another discussant from OOUTH group added similar point:

*“I think it should also be equitable, there should be equality that’s what I want to add.”* (Participant 3, female)

#### Early diagnosis and prompt treatment

Another discussant from OAUTH mentioned different dimensions to quality care. He emphasized the importance of early diagnosis and prompt treatment of patient in respectful manner.

*“When we talk about the quality of care, …. combination of factors that makes the patient to have high level of satisfaction. Number one, early diagnosis to be able to make your diagnosis, prompt treatment. That is one, then short interval that is short waiting time, then respectful interaction. Treat them with dignity and honour and then empathy, sympathy with minimal morbidity, with minimal complications so those are the ones that can easily be”* (Participant 26, male)

#### Provision of hospital equipment

Some discussants from all the groups expressed the importance of having needed hospital equipment in providing quality care. Lack of these useful materials can incapacitate care for the patient. A speaker from FMC buttressed this point with a scenario:

*“I will just give a scenario. A situation whereby the equipment is there, you have everything. But simple pregnancy test reagent is not available in the hospital. Pregnancy test, may be 10 naira or 20naira strip but the hospital is expected to provide it. you know that the frustration is both on the patient and even the health caregiver and you will be saying that simple thing like PT strip this hospital cannot provide so I believe that in terms of the working environment, it plays a major part.”* (Participant 18, male)

### Theme 3: Definition of personal wellbeing

A participant from UCH divided wellbeing into psychological and emotional dimensions. This participant made mention of ergonomics at workplace and its effect on physical wellbeing.

*“Just like when we try to refer to the World Health Organization's definition of health, personal wellbeing is beyond the physical health or physical well-being. There are other dimensions to it, even psychologically, emotionally and when we are now talking about, personal wellbeing in relation to what we are discussing, even the ergonomic implication of your workplace, your workstation, and how it eventually impacts on your physical well-being.”* (Participant 39, male)

#### Physical wellbeing

Most participants described their physical wellbeing in terms of physical comfort in their working environment.

*“And even if the environment is also not conducive ….you talk about ergonomics, sitting arrangement, back pain for the academics and things like that, this affects it too, the workspace will affect our physical health.”* (Participant 28, male)

*“…..even the ergonomic implication of your workplace, your workstation, and how it eventually impacts on your physical well-being. For example, you have come to work, you have by virtue of the relationship of your workstation to your own characteristics, they are incompatible. And then you end up having challenges even within your body physically, in terms of pain, in terms of whatever. So, eventually, even this impact on the physical well-being a good number of times, you end up with certain symptoms that you have to manage.”* (Participant 39, male)

#### Psychological wellbeing

A participant discussed the issue of unstable power supply which reduces zeal and passion to work.

*“Then talking about psychologically, you leave home, I'm going to work, I have this target, I want to do this today, I want to do that today. Sometimes, the most annoying part, maybe as you're coming in along the corridor, you see the light, and as you're just entering the door of your office like this, the light goes off that means certain plans are already distracted, you can't do what you have planned. And already psychologically, it depresses you, even emotionally, you get frustrated and things. So all these have impacted on me in the past.”* (Participant 39, male)

Another discussant shared how bearing the financial burden of the patients could lead to psychological torture for the caregiver.

*“At times the health care workers start contributing to be able to save life, this is very common in my unit. We need to save lives we need to provide for them. Like today now we've been contributing. It’s psychological torture for the care worker, you know what to do? You want to render the service but you are incapacitated.”* (Participant 17, female)

Another discussant shared how he was assaulted by a patient’s relatives.

*So coming to work every day before I leave home, I'll just say my wife pray for me so that I will come back home completely. I work in the accident and emergency, to be sincere that’s very fatal”.* (Participant 39, male)

#### Financial wellbeing

Another participant talked about financial constraints of patients as she had bought pampers/diapers for a patient before.

*“……..Because financially the patient is handicapped and the patient needs help from the system. ……it is for me to use personal money to do many things. ……we need to start contributing to be able to save life I've given a patient 40 Pampers, when she finished using this, she's asking me another one that ‘Madam, my pampers has finished’..”* (Participant 17, female)

### Theme 4: Quality of work life

The participants from all centers discussed extensively on their quality of work life.

#### Physical structures and environments

The participants were able to identify physical structures and environments within hospitals as the key dimension of quality of work life. Quite a number of the healthcare practitioners discussed about lack of offices and office furniture. Some said they had to be sleeping in their cars, walk around or stay at main university campus.

*“Like everybody have said, we are all in the same boat, as an assistant director in the laboratory, I do not have any office too. So aside from offices, like my colleagues said, basically, you come to work, there are things expected of you, we want to attend to our patients.”* (Participant 35, female)

*“And of course, we are also human beings. For example, you have your office, like I speak to you now. I don't have a very furnished office in this hospital. So I have to stay on campus, find my way down here, sitting inside my car and all that.”* (Participant 24, male)

Another participant from FMC made mention of always treating malaria because of mosquito bites from hospital environments and surroundings.

*“Apart from that, mosquitoes are always coming in because it's close to the gutter, so mosquitoes we just come and your legs would bite you, you know you're treating patient, you're always beating your legs, so I was always having malaria maybe every month or every 2 months. The people who do night too, we have to sleep to put whatever maybe mat or whatever and sleep.”* (Participant 15, female)

#### Inadequate salary and rewards

Participants here expressed their dissatisfaction at inadequate salary structure in healthcare system generally. A participant even said collecting hazard allowance of *₦*5000 was ridiculous.

*“So, and then to me as a person, that I'm able to feel fulfilled, happy and then have financial reward for my activities that the financial reward is commensurate to my input.”* (Participant 30, male)

*“Okay, let's look at inadequate salary. Salary. So, if you have you have to get this or say most of these things are not even provided in the hospitals like my nurse talked about you want to get anti-malaria, you have to come get it on your own, and the money is not just there. It's not enough, you can imagine ₦5000 for hazard allowance in hospital sector with.”* (Participant 22, female)

#### Delayed promotions

Participants weren’t happy with delayed promotions in their workplace. A participant reported being on the post of Assistant director for over nine years. Another participant who has also spent nine years on a post said all the management could do was to read and keep the certificates for them.

*“I want to talk about my own personal experience now, my last promotion was in 2012. This is 2021. So like they expect that if you are the SON type you should go to the university which I have done, my BSc, my master's everything you just keep reading and they're keeping the certificate for you, nothing is being done. Like the salary I was earning in 2012. That's what I'm still earning and we are expected to give quality care, well I don't know how I'm going to do that. Because I'm not happy. No promotion, nothing.”* (Participant 4, female)

#### Trainings and development

Participants were of the opinion that the world is evolving and there is a need to go for trainings and continuing professional development. A participants made a point of being turned back anytime the chance for professional development comes up. Another participant said he was at one point threatened to lose his job if he continued his PhD program.

*“And also about the postgraduate training, for example, I will not want to cite personal instances. But why I personally was embarking on a Ph. D program, we have to be called to the management level. And we had to be given a threat to either stop the program or we lose our job. And because I was just at that time bringing up a young family, I had to stop the program, the Ph. D program, to actually stay on the job. But that does not actually discourage me, I had to raise a lot of funds in foreign currency to pursue the PhD outside the country.”* (Participant 24, male)

## Discussion

This study described pattern and perception of wellbeing, quality of work life and quality of care offered by health professionals in southwest Nigeria. More than half of the participants reported poor personal wellbeing, quality of work life and quality of care offered to patients. This proportion is substantial as the wellbeing of health professionals is crucial for the provision of quality health care. Health professionals in Nigeria, like many African countries, reported poor personal wellbeing, work life quality and care compared with international health professionals [38, 40–43]. The African continent is facing healthcare service challenges including inadequate human resources, budget constraints in healthcare services, and poor leadership and management in healthcare [40] and these problems may be responsible for increased burnout among healthcare providers in sub-Saharan Africa which worsen poor performance in these construct [7]. This proportion of poor wellbeing reported in the present study is higher than the proportion of psychological distress reported among health care workers in Nigeria [20]. Though, both studies were from southwest Nigeria, the differences could be attributed to the hospitals’ bed-capacity where the participants are working. It is postulated that the higher the bed-capacity, the higher the workload which may precipitate poor wellbeing [44]. All the selected hospitals in the current study have higher bed-capacity than the hospital surveyed in the previous study. Culturally, it appears an average Nigerian would rather deny the psychological problems as these are not obvious to others but known to the person going through the problems [20]. However, our probes in the qualitative study revealed that some of the participants had psychological distress. Several factors contributing to poor wellbeing or quality of life among health professionals have been reported, ranging from personal factors to work-related factors [19, 20, 44, 45].

Adverse psychosocial work condition has been shown to results in poor quality of life among health care workers with imbalanced effort-reward i.e. high effort/low reward [45]. The results of our focus interviews buttressed this imbalanced effort-reward as adversely affecting their quality of life. Participants expressed their dissatisfaction at inadequate salary structure in healthcare system generally. A participant even said collecting hazard allowance of *₦*5000 ($12) was ridiculous. Participants were disappointed with delayed promotions in their workplace. A participant reported being on the post of Assistant Director for over nine years. Another participant who has also spent nine years on a post said all the management could do was to read and keep the certificates for them. A participant made a point of being turned back anytime the chance for professional development came up. Another participant said he was at one point threatened to lose his job if he continued his PhD program.

Demanding and high strained work environment was also suggested to contribute to poor wellbeing of health workers [44]. Nigeria, like most developing or low/middle-income nations, is characterized with inadequate health facility which usually results in demanding or strained work environment. This was corroborated by participants in focus interviews as participants discussed the issue of unstable power supply which reduces zeal and passion to work and eventually impact psychological wellbeing. Personal factors such as poor sleep have been reported to affect wellbeing and often lead to stressful life [19]. Many health professionals in Nigeria engaged in shift or call duty which may cut the hours of night sleep. There is the need to provide adequate amenities for the comfort of these health workers to reduce the stress of night duty. More so, most participants described their physical wellbeing in terms of physical comfort in their working environment.

Our data demonstrate unique patterns of personal wellbeing. It is noteworthy the gender difference in personal wellbeing of health professionals in this study in favor of females. This was corroborated by the results of logistic regression. Males were much less likely to report good personal wellbeing compared to females. Previous studies have documented females having better wellbeing/quality of life than males [20, 44]. Generally, in Nigeria context, men drink alcohol hazardously than women and this has been shown to impact psychological health [20]. This may account for better quality of life among female health professionals. Contrary to previous study from Nigeria, age was found to influence wellbeing [20]. With increase in age, the health professionals reported better wellbeing or quality of life. While the reason for this is unclear, it is likely that the older health professionals are near peak of their profession or have assumed leadership position which might have lifted work pressure off them. Our data suggest this as those with higher years of practice reported better personal wellbeing. Majority of people within the age ranges of 40–49 and 50–49 years constitute the bulk of professionals with ≥ 11 years of experience. This is plausible since nurses constituted the majority of the participants and on the average, it is presumed that they start their nursing career at the age of 20 years. There is a possibility that individuals of the same age range graduate from medical college at different ages and start working at different ages.

Many health professionals (especially the occupational therapists and radiographers) in this study reported poor quality of work life. This was in accordance with previous studies who reported poor or dissatisfied quality of work life among health professionals [38, 42, 43, 46]. The focus group results give an insight of what may likely contribute to the poor quality of work life. The participants identified physical structures and environments within hospitals as the key dimension of quality of work life. Quite a number of the healthcare practitioners discussed about lack of offices and office furniture. Some said they had to be sleeping in their cars, walk around or stay at the main university campus. This is detrimental as poor working environment is associated with burnout [7]. Conversely, investing in human resources for health e.g. providing enabling environment will not only results in good performance of health workers, but are necessary to the delivery of health services [47, 48], securing good working environment in addition to sufficient, adequately supported, equitably distributed, and well-performing health workforce will go a long way in achieving health goals and targets [46, 47]. It is noteworthy that occupational therapists and radiographers were the most poorly affected in the quality of work life. This is not clearly understood as there is little or no study primarily focusing on these groups assessing their quality of work life. However, it may be because of the inadequate human resources of these health professionals. These groups of health professionals are the least in terms of health professionals: population ratio in Nigeria [6].

The sociodemographic patterns of quality of work life are similar with previous study [46]. Health professionals with increased age, higher education and more years of practice or experience reported better quality of work life while increase in work volume decreases it [46]. It is apparent in Nigeria health sector that there is inadequate staffing and this may put more pressure on few health professionals available or increase their work volume and thus, affect their quality of work life. This calls for organization commitment in seeing to the welfare of health workers. This will not only improve quality of work life but also reduce turnover intensions [49].

Despite adequate knowledge of what quality of care entails exhibited by the participants, only few health professionals in this study reported providing quality care to the patients. It seems they have good sense of what good quality of care means as the focus group participants listed standard care; satisfaction of patient and healthcare provider; timely, efficient, excellent and equitable treatment; and holistic patient-centered care in their definition of quality care [50]. Our data suggest dimensions of quality of care that were responsible for low quality care reported. Amongst them are inadequate provision of care, materials, consumables and manpower; unsafe and unconducive environment; unequal and inequitable service; and late diagnosis and delayed treatment. These factors and others have been reported to compromise quality of health care provided [14, 40, 41]. These aforementioned factors buttressed deficiencies in the structure, process and outcome of care [40]. Nigeria like most African countries is facing health care service challenges such as inadequate budget allocation to health care services and human resources. While remedy to these inadequacies are not in sight, effort may be directed at balancing effort and reward of health professionals in Nigeria to motivate them in providing quality care as lack of reciprocity in terms of efforts and rewards impacts quality of care [51]. As noted earlier, poor wellbeing and quality of work life are contributing factors hampering quality care. Our data suggest that with a unit increase in personal wellbeing and quality of work life, there is 1.43 and 2.07 more likelihood to offer good quality of life, respectively. Therefore, key component to achieving good patient outcomes is having the right type and number of healthcare professionals with the right resources. Hence, adequate investment in infrastructure required for producing and retaining adequate numbers of health professionals is warranted [4].

This study has some policy implications. Addressing wellbeing of health professionals is critical and important for quality health care provision. Health organizations should direct effort at lowering job stress (which impact wellbeing) to reduce discordant care and strive to increase job resources to promote person-centered care[41]. Focus on health financing in Nigeria is also warranted. Priority should be given to provision of adequate and efficient facilities and enabling environment.

This study has some strengths and limitation. The major strength is the mixed methods design employed in the present study making it unique. The large number of included participants from different health disciplines added to the strength. However, the samples are from southwest Nigeria and not nationally representative. Thus, the results cannot be overgeneralized to all health workers in Nigeria. However, the sociodemographic characteristics of our study participants are very similar to previous studies of Nigerian health care workers at national and regional levels. The health care workforce in Nigeria is dominated by women who are mostly nurses and physicians [52], physiotherapists and radiographers are very few and mostly affected by the “brain drain” phenomenon as their services are in high demand abroad while fewer Nigerian universities offer the programmes [52]. In terms of age and educational levels, the majority of the Nigerian healthcare workers are young adults, this reflects the youth-based population of the country [52]; virtually all entry-level healthcare education in the country are university degree programmes. Therefore, our sample was representative of the study population. In the absence of nationally representative data, the unique pattern of personal wellbeing and quality of care of Nigerian health professional presented in this study may be an asset to health policy maker to improve health care delivery in Nigeria.

## Conclusion

More than half of health professionals reported poor quality of work life, quality of life and personal wellbeing which is influenced by personal and work-related factors. All these may have influenced poor quality of care reported despite exhibition of good knowledge on what quality of care entails. Effort directed towards improving personal wellbeing and quality of work life of health professionals may result in good health care delivery and quality outcomes.

## Data Availability

The datasets generated during and/or analysed during the current study are available from the corresponding author on reasonable request.

## Abbreviations

PW: Personal wellbeing
QoWL: Quality of work life
QoC: Quality of care
HPs: Health professionals
SWN: Southwest Nigeria
QoL: Quality of life
ILO: International Labour Organization
WHO: World Health Organization
GWB: General Wellbeing
HWI: Home-Work Interface
JCS: Job-Career Satisfaction
CAW: Control at Work
WCS: Working Conditions
SAW: Stress at Work
FMC: Federal Medical Centre
OOUTH: Olabisi Onabanjo University Teaching Hospital
OAUTHC: Obafemi Awolowo University Teaching Hospital Complex
UCH: University College Hospital
